# Can the recycling of LLIN reduce their coverage and use? Social, cultural and ethical aspects of LLIN life cycle management: exploratory qualitative data from Madagascar

**DOI:** 10.1186/1475-2875-11-S1-P77

**Published:** 2012-10-15

**Authors:** Ambinina Ramanantsoa, Rindra Rahenintsoa, Sarah Hoibak, Harilala Ranaivoharimina, Marthe Delphine Rahelimalala, Avotiana Rakotomanga, Alyssa Finlay, Joan Muela Ribera, Susanna Hausmann-Muela, Elizabeth Toomer, Koen Peeters Grietens

**Affiliations:** 1 Focus Development Agency, Antananarivo, Madagascar; 2 PASS International, Tessenderlo, Belgium; 3 Ministry of Health, Antananarivo, Madagascar; 4 Ministry of Environment, Antananarivo, Madagascar; 5 USAID-Deliver, Antananarivo, Madagascar; 6 CDC, Atlanta, USA; 7 Institute of Tropical Medicine, Antwerp, Belgium; 8 Shawnee International Health and Development, Shawnee, USA

## Background

There is growing awareness of the likely impact of increased numbers of LLIN on the environment, if not disposed of or recycled appropriately. The WHO and UNEP initiated a pilot study to identify and assess the feasibility of environmentally-sound and cost-effective options for collection, recycling and disposal of LLIN. In this context, several studies were conducted in rural Madagascar whereby 22,559 used bed nets were collected for recycling. A social science study was carried out to provide preliminary data on socio-cultural factors related to the collection and replacement of LLIN for disposal or recycling.

## Methods

Exploratory qualitative research was carried out following the pilot study in Betioky, Tsihombe, Fenerive Est and Ambanja, triangulating participant observation, interviewing and group discussions. Data analysis was a continuous, flexible and iterative process concurrent to data collection. Final analysis was carried out using NVivo 9.

## Results

It cannot be *a priori* excluded that the collection of LLIN that are being used in any form/way (sleeping, alternative and secondary uses) from households for recycling purposes can, under certain conditions, lead to lower LLIN coverage and use. Several factors account for this. (*i*) *Net preference.* LLIN use for malaria prevention is expected to decrease when the nets distributed after the collection do not meet local requirements, additionally leading to alternative uses. Consequently, community members were often not willing to hand over old nets before confirming that new nets were appropriate for their intended use. (*ii*) *Public/Private Sphere.* The collection campaign brings net use out of the private and into the public sphere, in certain cases leading to lower net use and presenting an additional problem for collection. Users stated feeling ashamed at having to present dirty, ripped or bad smelling nets in public. Such concerns can lead to users refraining from relinquishing nets and/or to reducing net use in order to keep nets presentable for future collection. (*iii*) *Net Lifecycle.* The economic value placed on nets, for both sleeping and alternative/ secondary uses, along with the sense of individual ownership of the nets, raises the question whether it is feasible to recycle nets during this stage of the net’s lifecycle. More so, given the fact that people will receive new nets based on epidemiological criteria and not in relation to their willingness to hand over used nets. Collecting nets at the stage of “waste” (when they are no longer used for any purposes) was locally more acceptable.

## Conclusion

The collection of used bed nets can be expected to be most feasible (i) for LLIN without locally perceived economic value, preferably at the stage of waste, (ii) when assuring users that net preference criteria are met by new LLIN, (iii) when the collection strategy is planned and appropriately explained to the community upon distribution of the LLIN. Given the possible concerns regarding net coverage and use, the collection strategy ought to be defined prior to net distribution and based on in-depth data on the local context.

